# Integrating molecular mechanisms and agronomic strategies for selenium biofortification in horticultural crops

**DOI:** 10.3389/fpls.2025.1714617

**Published:** 2025-12-03

**Authors:** Lixia Wang, Fahad Shafiq, Zheli Ding, Yingdui He

**Affiliations:** 1 Sanya Research Institute & Institute of Tropical Bioscience and Biotechnology, Chinese Academy of Tropical Agricultural Sciences, Haikou, China; 2 Department of Botany, Government College University Lahore, Lahore, Punjab, Pakistan

**Keywords:** biofortification, human nutrition, horticultural crops, selenium, selenoproteins

## Abstract

Selenium (Se) is an essential element for humans. Biologically, it is incorporated into selenoproteins, which play crucial roles in thyroid hormone metabolism, immune system regulation, and antioxidant capacity. However, Se deficiency is a global health concern, affecting over 1 billion individuals, and the production of Se-fortified crops is inevitable. As a way forward, the biofortification of horticultural crops could improve Se nutrition. For plants, Se is a beneficial element, and crops exhibit significant variation in their ability to uptake Se which is also influenced by soil pH. Therefore, this review focuses on recent advancements in Se biofortification technologies for horticultural crops, including soil and foliar application methods, and explores the physiological processes and genetic mechanisms underlying Se uptake, transport, and assimilation in these crops. Furthermore, the effectiveness of different Se-salts in the regulation of Se levels in crops has been discussed, with special emphasis on the mechanisms involved.

## 1 Introduction to selenium nutrition

Selenium (Se) is a member of the chalcogen family and is required for key cellular physiological functions in humans (Banuelos et al., 2023). Trace quantities of Se in the diet are critical for disease prevention because they support the immune system (Lan et al., 2024). In contrast, its deficiency is linked to Keshan disease, a cardiovascular disease primarily affecting children and females, and bone disease, which is a chronic, degenerative osteoarticular disorder (affects bones and joints (Rayman, 2012). Other health conditions include thyroid disorders, autoimmune and neurodegenerative diseases, and cancer, which can lead to death (Ekumah et al., 2021; Duță et al., 2025). It is estimated that approximately 0.5–1 billion individuals exhibit inadequate Se uptake, which is a serious health concern (Jones et al., 2017; Dinh et al., 2019). Alternatively, adequate Se uptake boosts immunity in both animals and humans. In China, agricultural lands are deficient in Se, and approximately 60% of the Chinese population suffers from inadequate dietary Se intake, which is a serious health concern (Liu et al., 2021a).

Selenium is a beneficial element that affects plant growth under certain environmental conditions. Human selenium nutrition is primarily linked to the consumption of plant-based foods, with fruits and vegetables serving as important sources. However, its uptake is dependent on the plant species, soil type, and available forms of Se. Naturally, Se occurs in both organic and inorganic forms, depending on soil pH, redox status, and mineral composition (Qu et al., 2023). In general, selenite (SeO_3_^2-^) tends to predominate in acidic and anaerobic soils, whereas selenate (SeO_4_^2-^) is abundant in soils with high pH. In addition, Se can exist in the soil in an organic form, such as selenomethionine (SeMet), which ranges from 15-45% of the soluble Se fraction, depending on the soil type (Yamada et al., 1998; Supriatin et al., 2015). It is pertinent to mention that SeMet only represents a very small fraction of organic Se of the total available Se. Therefore, bioavailable Se in soil is predominantly present in inorganic forms, such as selenate and selenite. Seleniferous soils can exhibit Se concentrations of up to 10 µg g^-1^ (McNeal and Balistrieri, 2015). For non-seleniferous soils, Se in soil typically ranges between 0.01 and 2 µg g^-1^, averaging 0.4 µg g^-1^ (Wang et al., 2022a). It is essential to clarify that Se deficiency is also prevalent in most global soils, but this does not imply that all non-seleniferous soils are deficient. Seleniferous soils (naturally enriched in Se) provide adequate selenium without deficiency risks; however, non-seleniferous soils (the majority) typically exhibit selenium deficiency, although localized variations exist.

As previously mentioned, the uptake of Se by plants is complex and is influenced by several factors. Both phosphorus (P) and sulfur (S) compete with plant root Se uptake owing to their structural similarities and shared uptake pathways. Furthermore, different elements compete with Se for translocation, thereby limiting its uptake (Ribeiro et al., 2016; Gui et al., 2022). In addition, plants have a very limited capacity to absorb Se from the soil, which makes Se nutrition challenging (Nie et al., 2024). The current review focuses on recent developments in the Se biofortification of horticultural crops, including fruits and vegetables. This study presents an in-depth analysis of the key physiological processes involved. This will pave the way for future research on the biofortification of crops cultivated under a wide range of environmental conditions.

## 2 Research progress on selenium-enriched foods

The Chinese Society of Nutrition recommends a daily selenium intake of 60 µg for adults, whereas the World Health Organization (WHO) recommends a daily selenium intake of 60-200 µg for healthy adults, with a tolerable upper intake level of 400 µg (Kipp et al., 2015; Yang et al., 2021). Approximately 15% of the global population exhibits a lower Se intake (WHO, 2004), over 1 billion individuals worldwide suffer from Se deficiency, and 70% of Chinese soils are Se deficient (Stoffaneller and Morse, 2015). In a comprehensive review, Zhao et al. (2024) reported significant regional variations in soil Se levels across different Chinese provinces. The study concluded that Se concentrations in soils were also dependent on soil parent material, with high Se contents in siliceous rocks; however, plant Se uptake was not dependent on total soil Se concentration (Zhao et al., 2024). Therefore, the Se levels in plants are insufficient to meet human requirements (Broadley et al., 2006; Dijck-Brouwer et al., 2022). In general, plants vary in Se uptake capacity and can be grouped as non-accumulators (<100 μg g^-1^ DW), accumulators (100-1000 μg g^-1^ DW), and hyper-accumulators (>1000 μg g^-1^ DW). Approximately 100 dicot species have been identified as Se hyperaccumulators across different families (White, 2016). Moreover, the uptake, translocation, and assimilation of different forms of Se vary among different plant species. The most abundant form of Se in edible seeds and vegetables is SeMet. However, Se non-accumulator species metabolize inorganic selenium into organic forms, primarily SeMet in grains and legumes; therefore, selenate is not the terminal product (Smrkolj et al., 2007; Shao et al., 2014; Huang et al., 2017).

Selenium is beneficial to plants in trace quantities, and Se biofortification of crops has gained particular attention in recent years. Horticultural crops can serve as a source of Se to counter Se malnutrition in humans; however, this is a complex approach that depends on multiple factors. Various researchers have reported the beneficial effects of Se application on plants, and considerable success has been achieved in terms of Se biofortification in crops. In cereals, the Se content can be enhanced by foliar application of seleniferous compounds (Radawiec et al., 2021). For instance, seleno-cyanoacetate application causes the accumulation of selenomethylcysteine (SeMeCys) in brown rice (Yuan et al., 2023). In addition, the combined application of SeMet and P enhanced selenocysteine (SeCys) levels in winter wheat (Hu et al., 2023). It has also been reported that SeCys are more effective and enhance Se translocation in grains and further assimilation into organic compounds (Liu et al., 2023a). Exogenous Se also improves the crop quality characteristics of wheat and maize (Broadley et al., 2010; Zahedi et al., 2019). In fruits, Se foliar spraying improved the Se content in apples and oranges (Wojcik, 2023; Wang et al., 2024). Improvements in the quality characteristics of strawberries and their Se content by exogenous Se have also been reported (Zahedi et al., 2019). In addition, biofortification of vegetables, legumes, forages, and medicinal plants has been performed (reviewed by Huang et al., 2024b). The foliar application of sodium selenate and Se nanoparticles (10 mg L^-1^) improved the biochemical composition of tomato fruits, including protein-related quality parameters (Shiriaev et al., 2023). Similarly, potato treated with nano-Se fertilizer exhibited upregulation of the enzyme activities of GSH, POD, PPO, SOD, and PAL and increased vitamin C and protein content in tubers (Liu et al., 2025). Similarly, soil and foliar application of exogenous Se improved citrus quality (Wang et al., 2024). Four-time application of Na_2_SeO_4_ at 15 g ha^−1^ spray^−1^ improved Se content in fruits up to 12 folds (Wójcik, 2024). A summary of different studies related to the biofortification of different horticultural crops is presented (**Table 1**).

**Table 1:** Status of selenium fortification in food crops (vegetables) across the globe.

Sr. No.	Species	Study objectives/plant species	Findings	Conclusive remarks	References
1	Wheat (*Triticum aestivum* L.), corn (*Zea mays* L.), rice (*Oryza sativa* L.), broccoli (*Brassica oleracea* var. italica), garlic (*Allium sativum* L.), onion (*Allium cepa* L.), leek (*Allium ampeloprasum* var. porrum), Indian mustard (*Brassica juncea*), and hyperaccumulators (e.g., *Astragalus bisulcatus*)	To review strategies for selenium (Se) biofortification in staple crops and Se-accumulating plants to address global Se malnutrition.	Agronomic biofortification: Foliar Se application increased Se concentrations in wheat (42-67 µg kg^-1^) and corn (19-36 µg kg^-1^); rice Se content varied widely (5–1370 ng g^-1^) depending on soil type. Genetic biofortification: Overexpression of genes (APS, SMT, CgS) enhanced Se accumulation and tolerance in crops (e.g., Indian mustard, tomato). Soil dynamics: Se bioavailability is higher in selenate-treated aerobic soils than selenite-treated anaerobic soils; water management (e.g., drainage) optimizes grain Se retention. Health/economic impact: Biofortified crops reduce Se deficiency-related diseases (e.g., Keshan disease) and healthcare costs, improving productivity.	Se biofortification (via agronomic practices, genetic engineering, and soil management) is critical for combating global Se malnutrition, especially in rice-dependent regions. Future priorities: Enhance Se bioavailability, optimize genetic traits for Se accumulation in edible parts, and address challenges (e.g., consumer acceptance, heavy metal co-uptake). Multidisciplinary collaboration and policy frameworks are essential for scaling biofortification and ensuring long-term food/nutrient security	Ranawake et al., 2025
2	Maize (*Zea mays* L.), millet (*Pennisetum glaucum*), sorghum (*Sorghum bicolor* L.), and cassava (*Manihot esculenta*)	To assess the impact of Se-deficiency on human health in Sub-Saharan Africa. Evaluate biofortification strategies (especially agronomic biofortification) to improve selenium intake through local crops. Understand soil-plant-human transfer pathways of selenium in the region.	Selenium deficiency is widespread across Sub-Saharan Africa, contributing to increased vulnerability to diseases, including viral infections and immune dysfunction. The selenium content in crops is highly variable due to soil type, climate, and agricultural practices. Biofortification (adding selenium to fertilizers) has shown promising results in increasing crop selenium content, particularly in Ethiopia and Malawi; Agronomic biofortification is more immediately effective compared to genetic methods or food fortification.	Selenium deficiency is a public health challenge in Sub-Saharan Africa, deeply linked to soil quality and crop composition; Targeted interventions, especially agronomic biofortification, offer a cost-effective and scalable solution; Policymaking should prioritize integrated strategies involving agriculture, health, and nutrition to address selenium malnutrition.	Botha et al., 2024
3	Teff (*Eragrostis tef*), wheat (*Triticum aestivum* L.), and maize (*Zea mays* L.)	To estimate the health burden of selenium deficiency in Ethiopia. To assess the cost-effectiveness of agronomic biofortification (applying selenium fertilizers) in staple crops.	Selenium deficiency contributes significantly to disease burden, particularly by weakening immune function and increasing susceptibility to infections. Agronomic biofortification could significantly reduce disability-adjusted life years (DALYs) at a relatively low cost. Regions with very low soil selenium are most at risk and benefit the most from intervention.	Agronomic biofortification is a cost-effective intervention for improving selenium status in Ethiopia. Targeting high-risk areas and scaling up fertilization practices could yield meaningful public health improvements. Integrating this strategy into broader agricultural and nutrition programs is highly recommended.	Abdu et al., 2023
4	Focus is on plants role in Se management	Review selenium dynamics in agroecosystems: uptake, transport, toxicity, and sustainable management. Evaluate phytotechnologies as eco-friendly strategies to manage Se levels.	Traditional Se remediation methods are expensive and environmentally harmful, making phytotechnologies a more sustainable alternative. There is a need for interdisciplinary collaboration to better understand Se dynamics in agricultural systems.	A sustainable balance is needed between correcting Se deficiency and avoiding toxicity. Phytotechnologies offer viable solutions, but require more interdisciplinary research and field optimization. The review highlights the potential for eco-friendly, cost-effective strategies to manage Se in agricultural systems and improve public health.	Somagattu et al., 2024
5	Rice (*Oryza sativa* L.)	To assess Se-biofortification strategies focusing on market surveys, field sampling, and controlled experiments.	Global rice Se concentrations were 0.079 mg kg^-1^. 70% of East Asian paddy soils have insufficient Se bioavailability to meet global rice Se standards. Soil iron oxide content influence rice Se enrichment. Activating native soil Se (vs. exogenous addition) and water management are effective biofortification strategies.	Prioritize activating native soil Se and optimizing water management rice Se-enrichment Address low Se bioavailability in soils and leverage farmland-to-table insights to combat Se hidden hunger. Emphasize region-specific strategies and future research on bioavailability optimization and agronomic practices.	Ma et al., 2024
6	Winter wheat *Triticum aestivum* L.	To enhance selenium (Se) content in winter wheat grains through genetic biofortification to address human Se deficiency, focusing on breeding and genetic engineering approaches.	Genetic biofortification is more sustainable than agronomic methods. Wild wheat relatives (e.g., T. dicoccoides) exhibit higher Se accumulation potential. QTLs for Se content mapped on chromosomes 3D, 5B, and others; chromosome 3D shows significant effects on Se uptake via root traits. Transgenic approaches (e.g., introducing SMT gene) increase Se accumulation but face regulatory/ethical challenges.	Genetic biofortification offers a promising long-term solution to improve Se content in wheat. Further research is needed to validate QTLs, explore wild relatives, and address safety concerns in transgenic strategies. Combining genetic breeding with microbial-mediated biofortification may optimize outcomes.	Sunic and Spanic, 2024
7	Various crop plants (e.g., cereals, vegetables)	To investigate optimal conditions for agronomic biofortification of iodine and selenium in crop plants to address human deficiencies.	Agronomic biofortification effectively increases iodine/selenium in crops. Optimal application methods (e.g., foliar/soil) and doses are critical for success. Awareness among producers/consumers is essential for implementation. Limited data exist on bioavailability and long-term efficacy of biofortified foods.	Agronomic biofortification shows promise to combat iodine/selenium deficiencies, but standardized protocols and human bioavailability studies are needed. Public education and collaboration between stakeholders are key for scalability.	Oztekin and Buyuktuncer, 2024
8	Cereals (e.g., maize (*Zea mays* L.), wheat (*Triticum aestivum* L.), rice (*Oryza sativa* L.) Legumes (e.g., lentil (*Lens culinaris*), soybeans (*Glycine max* L.) Vegetables (e.g., lettuce (*Lactuca sativa*), tomato (*Solanum lycopersicum*) Forages (e.g., alfalfa (*Medicago sativa*), ryegrass (*Lolium perenne*) Medicinal plants (e.g., *Atractylodes macrocephala*)	To evaluate key factors influencing selenium (Se) biofortification via fertilization and optimize Se application strategies to enhance Se content in crops while minimizing yield reduction. Focused on cereals, legumes, vegetables, forages, and medicinal plants.	Plant type and Se fertilization rate are primary factors affecting Se content and yield. Se content increases with fertilization up to thresholds (164 g ha^-1^ for cereals, 103 g ha^-1^ for forages). High Se doses reduce yields, particularly in cereals. Foliar application is more effective than soil application; selenate and selenite show similar efficacy. Initial soil Se content, organic carbon, pH, and climate (temperature, precipitation) also influence outcomes.	Balanced Se fertilization strategies (80–100 g ha^-1^) are critical for biofortification without yield loss. Long-term ecological monitoring and bioavailability studies are needed. Plant type-specific approaches and consideration of soil/climate factors can optimize Se biofortification.	Huang et al., 2024
9	Major food crops (e.g., cereals, legumes, vegetables).	To evaluate the role of nanoparticles (NPs) in enhancing biofortification of food crops (e.g., cereals, legumes) to address malnutrition, focusing on NP-based strategies for increasing micronutrients (Zn, Cu, Fe, Se) in crops while minimizing environmental and health risks.	Nanotechnology is an environmentally friendly technique for enhancing nutritional value in crops. Nutrient-based nanoparticles (NPs) can increase essential micronutrients in food crops. NPs of zinc, copper, iron, and selenium are effective in boosting human immune systems against viral infections and health threats. Traditional methods like chemical fertilizers have drawbacks, including environmental degradation and reduced food quality. Nanoparticles offer a sustainable alternative to traditional fertilizers, improving both crop yield and nutritional quality.	Nanotechnology offers sustainable biofortification solutions to combat malnutrition by improving crop nutrient content and safety. Integrating genetic/agronomic approaches with NP-based strategies can optimize crop yield, quality, and human health outcomes. Further research and adoption are needed to scale these methods globally.	Zahid et al., 2025
10	Rice (*Oryza sativa* L.), with regional cultivars like Longgeng, Jingliangyou 534, Zhongzheyou 1, and Yixiangyou 2115.	To evaluate selenium (Se) distribution in Chinese rice paddy soils, analyze cultivation practices affecting Se biofortification in rice, and propose strategies to enhance Se content in rice grains.	China’s rice-growing areas show varying degrees of selenium deficiency. Selenium fertilizer application increases Se in rice grains and improves yield and quality. Nitrogen fertilizer enhances selenium content, while phosphorus and potassium fertilizers have less impact. Water management practices, especially alternate wetting and drying (AWD) irrigation, significantly affect selenium bioavailability. Different rice varieties vary in selenium content due to genetic differences.	Region-specific water/fertilizer management and varietal selection can enhance rice Se content. Prioritize breeding Se-enriched, high-yielding rice varieties and long-term monitoring of environmental impacts. Integrating agronomic practices and genetic improvements is critical for sustainable Se biofortification in China	Li et al., 2024
11	Wheat (*Triticum aestivum* L.), rice (*Oryza sativa* L.), soybean (*Glycine max*), tomato (*Solanum lycopersicum*), lettuce (*Lactuca sativa*), and garlic (*Allium sativum*) etc.	To evaluate the role of selenium (Se) biofortification via effective microorganisms (EM) in enhancing crop tolerance to abiotic stresses (drought, salinity, heavy metals) and improving nutritional quality. Focused on leveraging microbial interactions to increase Se accumulation in crops like wheat, rice, and vegetables.	Selenium is essential for human health, acting as an antioxidant and supporting immune function. Selenium biofortification can enhance crop nutritional quality and help address micronutrient deficiencies. Effective microorganisms (EM) can promote selenium uptake in plants and improve stress tolerance. The application of selenium through foliar sprays, soil fertilizers, and nanoparticles shows promise for increasing crop yields and quality. Excessive selenium can lead to toxicity in plants and humans, highlighting the need for careful management.	Selenium biofortification via EM offers a sustainable strategy to address micronutrient deficiencies and abiotic stress in crops. Integrating microbial inoculants (e.g., selenobacteria) with optimized Se application (foliar/nano-forms) enhances crop resilience and nutritional value. Future research should focus on microbial mechanisms, environmental impact assessment, and regulatory frameworks to balance benefits and risks. This approach aligns with global goals for food security and sustainable agriculture	Yadav, 2024
12	Maize (*Zea mays* L.), wheat (*Triticum aestivum* L.), soybean (*Glycine max*), sunflower (*Helianthus annuus* L.), and forage grasses	To assess the selenium (Se) status in soils, crops, livestock, and humans across Southern Africa and evaluate biofortification strategies to address deficiencies. Focused on staple crops (maize, wheat) and forages as primary dietary sources of Se for both livestock and humans.	Selenium levels in Southern African soils are generally low, leading to selenium-deficient crops, particularly maize, which is a staple food. Selenium deficiency is widespread in human and animal populations, contributing to health issues such as impaired immune function and increased susceptibility to diseases. Biofortification of crops through selenium fertilization can significantly improve selenium content in grains and pasture. Selenium supplementation in animal feed enhances selenium levels in animal products, benefiting both animal health and human nutrition.	Southern Africa faces critical Se deficiencies in soils, crops, and populations, necessitating urgent intervention. Biofortification of staple crops (e.g., maize) via Se fertilizers or transgenic breeding is a viable solution. Integration of microbial-mediated strategies and policy support (e.g., Finland’s model) could improve Se status sustainably. Further research is needed to optimize biofortification methods, monitor long-term impacts, and address socioeconomic barriers to implementation.	Chilala et al., 2024
13	Common bean (*Phaseolus vulgaris* L.)	To evaluate the effects of Se-enriched urea and ammonium sulfate on agronomic, physiological, and nutritional traits	Se-enriched fertilizers enhanced seed yield by improving key physiological processes	Se-enriched urea was found to be more effective in increasing Se concentrations in common bean seeds, likely due to increased Se availability in the soil near the urea granule.	Namorato et al., 2025
14	Barley (*Hordeum vulgare* L.)	To enhance selenium (Se) content through exogenous application for addressing Se deficiency in Tibetan Plateau diets.	Exogenous Se fertilizers improved barley Se fraction which was mostly accumulated in the aleurone layer outside the inner endosperm. Pearling processing led to significant losses of Se and other micronutrients, with the removal of pearling fractions P6 resulting in a 24.10% loss of Se.	Se biofortification combined with optimized processing (e.g., reduced pearling, thermal treatments) effectively maintains Se levels in highland barley within recommended ranges, offering a sustainable strategy to combat Se deficiency in Se-deficient regions like the Tibetan Plateau. Further research on processing–nutrient interactions is needed to maximize nutritional benefits.	Li et al., 2025
15	Cow pea (*Vigna unguiculata* (L.) Walp.	The study aimed to determine the genotypic variability of cowpea in response to selenium (Se) application to develop agronomic biofortification strategies. The plant species studied was cowpea.	Genotypic variation: 20 cowpea genotypes exhibited differences in yield and nitrogen efficiency. Se fertilization: Soil/foliar application of sodium selenate (0–150 g ha^-1^) influenced physiological traits (photosynthesis, antioxidant activity) and nutrient accumulation. Nutrient distribution: Se biofortification increased essential/beneficial elements in grains while monitoring antinutrients. Responsive genotypes showed higher Se accumulation without yield penalties.	The results may contribute to the selection of responsive cowpea genotypes for biofortification programs and the determination of safe Se doses. This could help combat nutritional deficiencies through agronomic strategies, potentially addressing hidden hunger by enhancing the nutritional content of cowpea grains.	Lanza, 2024
16	*Allium* species *viz.* Garlic (*Allium sativum* L.), onion (*Allium cepa* L.), leek (*Allium ampeloprasum* L.), chives (*Allium schoenoprasum* L.), and shallot (*Allium cepa* var. *aggregatum*)	To evaluate the potential of selenium (Se) biofortification in *Allium* crops for functional food production, focusing on genetic traits, Se chemical forms (selenate, selenite, nano-Se), application methods (soil/foliar), sulfur (S) and arbuscular mycorrhizal fungi (AMF) interactions, and hormonal regulation. Additionally, to explore the development of Se-enriched functional foods (e.g., bread with leek powder, microgreens, black garlic) and optimize cultivation strategies to enhance anti-carcinogenic properties.	The study found that Se treatment positively influenced the accumulation of secondary metabolites and plant yield in *Allium* species.	*Allium* crops exhibit high potential for Se biofortification, but responses vary due to genotype and environmental factors. Optimization strategies (e.g., biochar, Si compounds, AMF inoculation) and novel Se forms (nano/organic) are needed to enhance Se accumulation and stress resilience. These advancements could position Se-enriched *Allium* products as functional foods with medicinal value, addressing nutritional deficiencies and supporting sustainable agriculture. Future research should prioritize cost-effective, scalable methods and long-term safety assessments.	Golubkina et al., 2024b
17	Wheat (*Triticum aestivum* L.), barley (*Hordeum vulgare* L.), rice (*Oryza sativa* L.), corn (*Zea mays* L.), oilseed rape (*Brassica napus* L.), and leafy vegetables e.g., pakchoi (*Brassica rapa subsp. Chinensis*), and Se hyperaccumulators like *Stanleya pinnata*.	To enhance selenium (Se) transfer from soil to plants via optimized agronomic strategies, focusing on improving Se bioavailability in crops (e.g., cereals, leafy greens) to address human dietary deficiencies. The review emphasizes mechanisms of Se mobilization, soil–plant interactions, and metabolic pathways in plants.	Selenium in soil exists in various chemical forms, with plants absorbing selenite and selenate. Key factors influencing Se bioavailability include redox potential, pH, organic matter, moisture, and microbial activity. Strategies to boost Se transfer involve adjusting these factors and promoting beneficial microbes. Plants can convert inorganic Se into organic forms beneficial for humans. Foliar Se application is effective but requires careful timing and concentration.	Integrated soil management (e.g., redox/pH optimization, microbial inoculation) and targeted Se application (foliar/soil) can significantly improve Se biofortification. Challenges include complex soil-plant interactions and variability in Se speciation. Future research should prioritize genotype-specific approaches, microbial consortia, and advanced technologies (e.g., nanotechnology) to maximize Se transfer and sustainability, ensuring Se-rich crops contribute to global nutritional security	Liao et al., 2024
18	Rice (*Oryza sativa* L.)	To evaluate the effects of Se on rice grown in Cd-contaminated soil	The Se supplementation significantly reduced Cd accumulation in rice roots, shoots, and grain by 16.3%, 24.6%, and 37.3%, respectively. Additionally, Se influenced Cd accumulation by regulating expression of Cd transporter genes leading to phytoremediation.	Se supplementation is a promising strategy to simultaneously reduce Cd toxicity and biofortify rice with Se, particularly in lightly contaminated soils. Future research should optimize Se application methods, explore long-term environmental impacts, and investigate genotype-specific responses to maximize efficacy and sustainability.	Huang et al., 2024

## 3 Technical means of selenium nutrition enhancement

It is believed that higher plants lack the ability (e.g., the selenocysteine insertion machinery guided by the UGA codon) to incorporate Se into proteins in a specific manner. It is believed that Se can be non-specifically incorporated into proteins by replacing cysteine or methionine residues (Cone et al., 1976). Selenocysteine (SeCys) insertion in proteins is dictated by the UGA codon (a termination signal), which requires SeCys insertion sequence (SECIS) elements (Berry et al., 1991). A selenocysteine insertion sequence and a pair of tRNA-Sec have been identified in the mitochondrial genome of cranberries (Fajardo et al., 2014). Although the exact mechanism remains unclear, it has been identified that an elongation factor, EEFSEC, recognizes the extended variable arm of tRNA^[Ser]Sec^, which also contains some modifications in its anticodon loop, including i6A (N6- isopentenyladenosine) at position 37 and two forms of 5-methoxycarbonylmethyluridine (mcm^5^U and mcm^5^Um) at position 34 (see detailed review by Chavatte et al., 2025). Nonetheless, an efficient way to improve Se nutrition in crops and promote its assimilation into the human diet is to use Se in soil or as a foliar spray for horticultural crops. Also, the Se-enriched fertilizers have been used globally for a long time (Ogunsuyi et al., 2025). In this section, we provide in-depth details regarding the pros and cons of Se-soil and foliar-based applications to draw parallels between them and to highlight which approach is better.

### 3.1 Insights into Se application in soils and associated considerations

To address Se deficiency in humans, the application of Se-rich fertilizers to soil is recommended. However, the direct addition of Se to soil does not ensure the success of Se-enriched crops because of several factors. Plant root Se uptake is primarily dependent on soil physicochemical properties, such as pH, Eh, and Se form applied.

Se naturally exists as selenide, elemental selenium, thioselenate (Se_2_O_3_^2-^), selenite (SeO_3_^2-^), and selenate (SeO_4_^2−^), corresponding to the five oxidation states of Se^2-^, Se^0^, Se^2+^, Se^4+^, and Se^6+^, respectively (Guo et al., 2023). Additionally, soils may contain various forms of organic Se, such as SeMet, SeCys, and MeSeCys (Thiry et al., 2012). However, only SeO_4_^2-^ and SeO_3_^2-^ are the dominant Se oxyanions in the soil solution absorbed by plant roots (Dinh et al., 2019), whereas other Se species can be adsorbed onto Fe and Mn oxides or even organic matter, making them unavailable to plants (Weng et al., 2011; Lyu et al., 2021; Guo et al., 2023). Nonetheless, it has also been well established that plants cultivated in alkaline soils prefer SeO_4_^2-^ (+6 oxidation state), whereas those in acidic soils tend to take up SeO_3_^2-^ (+4 oxidation state) (Elrashidi et al., 1987). Plants take up other forms of Se, including organoselenium species, SeMet, and SeCys (König et al., 2012). Similarly, in wheat plants cultivated in acidic soils, selenite uptake is preferred over selenate uptake (Kikkert and Berkelaar, 2013).

Therefore, SeO_4_^2-^ becomes more bioavailable in oxic soils, whereas SeO_3_^2-^ dominates anaerobic acidic soils (Mikkelsen et al., 1989; Bassil et al., 2018). Furthermore, under reduced conditions (Eh < -200 mV), SeO_3_^2-^ can be converted to insoluble Se species (Se^0^ and Se^2-^), thereby reducing its bioavailability (Nakamaru and Altansuvd, 2014; Bassil et al., 2018). This also tightly binds SeO_3_^-^ and locks it into the soil profile, causing Se deficiency in crops (Peak and Sparks, 2002; Kumar et al., 2011). In alkaline soils, SeO_3_^2-^ can be oxidized to SeO_4_^2-^ (Dinh et al., 2019). It has been estimated that approximately half of the total Se concentration in soil is locked with clay minerals and organic matter, making it unavailable to plants (Weng et al., 2011; Qin et al., 2012). Soil amendment with either SeO_4_^2-^ or SeO_3_^2-^ causes a very high leaching rate (Zhai et al., 2019). In addition, depending on the soil pH, the available SeO_3_^2-^ can exist as H_2_SeO_3_ (at pH 3), HSeO_3_^−^ (at pH 5), and SeO_3_^2−^ (at pH 7) (Hopper and Parker, 1999; Wang et al., 2019). It is pertinent to mention that phosphate transporters and anion channels contribute to the uptake of HSeO_3_^−^ and SeO_3_^2−^ in plants, which exist at pH 5 and pH 7, respectively (Zhang et al., 2014; Zhao et al., 2024). In contrast, SeO_4_^2-^ uptake occurs via sulfate transporters, and S-starvation contributes to a 10-fold increase in Se uptake (Li et al., 2008). However, the Se-hyperaccumulator *Stanleya pinnata* did not show any competitive inhibition, even at a 100-fold excess of sulfate (El Mehdawi et al., 2018). In contrast, Se uptake in *Brassica juncea* was reduced by 40%, and in *Stanleya elata*, it was reduced by 100% (El Mehdawi et al., 2018). A hydroponic study revealed that a lower supply of S increased Se content and root-to-shoot translocation in *Brassica napus* by 06 times (Ren et al., 2023). Similarly, findings of field experiments from Rothamsted, UK revealed upregulation of sulfate transporters (SULTR1;1 and SULTR4;1) under reduced S supply, which contributed to 07-fold Se increase in wheat grain (Shinmachi et al., 2010). Considering that the soil application of Se does not seem to be a plausible approach because most of the uptake would be dependent on soil physiochemical attributes, it is challenging. A list of previous studies in which Se was applied directly to the soil and its subsequent contribution to Se biofortification is presented (**Table 2**).

**Table 2:** Biofortification of Se in horticultural crops by soil/root zone application - recent reports.

Sr. No.	Plant species/part	Se compound applied	Se concentration applied	Mode of application	Physiological effects/ Se biofortification	Fold change (compared to control)	References
1	Chives (*Allium schoenoprasum*)	Na_2_SeO_3_	0, 20, 40, 80, and 160 mg L^−1^	Soil application (three times)	- Regulation of phenylpropanoid pathway - 40.2 mg kg^-1^ DW Se accumulation in response 160 mg L^-1^ application	2010-fold	Chen et al., 2025
2	Citrus (*Citrus reticulata*)	Na_2_SeO_3_	0 and 100 mg L^-1^	Soil application	- 9.25 ng g^-1^ Se fruit - 92.1 ng g^-1^ Se in leaf	2.74-fold 1.63-fold	Wang et al., 2024
3	Onion (*Allium cepa* L.)	Na_2_SeO_4_	0, 10, 25, 50, 100 and 200 g ha^-1^	Soil application	- Increase in onion yield up to 99 kg ha^-1^ - 0.600 mg kg^-1^ Se in bulb	60-fold	Machado et al., 2024
4	Komatsuna (*Brassica rapa* var. *perviridis*)	2-iminoselenazolidin-4-ones (ISeA)	0 and 10 mg L^-1^	Root zone	- 34.7 mg kg^-1^ Se in leaves	35-fold	Semenova et al., 2024
5	Chard (*Beta vulgaris* subsp. *cicla*)	2-iminoselenazolidin-4-ones (ISeA)	0 and 10 mg L^-1^	Root zone	- 23.8 mg kg^-1^ Se in leaves - 55.6 mg kg^-1^ Se in petioles	24-fold 56-fold	Semenova et al., 2024
6	Sweet cherry (*Prunus avium*)	Na_2_SeO_4_	250 and 500 g ha^−1^	Soil application	- Leaf Se concentrations up to 1.178 mg kg^-1^ (two-year average) - Fruit Se up to 2.45 µg 100 g^-1^ FW (two-year average) - No leaf or fruit injury by Se application	27.3-fold 8.16-fold	Wójcik, 2024
7	Tomato (*Lycopersicum esculentum* L.)	Na_2_SeO_4_ Na_2_SeO_3_	0, 1 & 5 mg kg^-1^ each	Soil application	- Selenate increased tomato yield and Se accumulation in tomato root (28.5 mg kg^-1^), stem (16 mg kg^-1^), leaves (34 mg kg^-1^) and in fruits. - Selenite caused Se accumulation in tomato root (7.5 mg kg^-1^), stem (2 mg kg^-1^), leaves (6.2 mg kg^-1^) and in fruits.	18.5-fold (root) 11.3-fold (stem) 28.6-fold (leaf) 12.3-fold (root) 1.33-fold (stem) 4.13-fold (leaf)	Kang et al., 2024
8	Cowpea (*Vigna unguiculata*)	Sodium selenate	0, 10, 25, and 50 g Se ha^−1^ combined with S (S (0, 15, 30, and 60 g ha^−1^)	Soil application	- In the absence of S, leaf Se concentrations went up to 2.55 mg kg^-1^ (average two-year) - A two-year average seed Se contents were 1.45 mg kg^-1^	8.5-fold change 4.83-fold change	Silva et al., 2023
9	Rocket (*Eruca sativa*)	Na_2_SeO_4_	0, 2.6, 3.9, or 5.2 μmol L^−1^	Root zone/Hydroponics	- Leaf Se contents up to 55 µg g^-1^ DW in response to 5.2 μmol L^−1^	110-fold change	Francini et al., 2023
10	Lettuce (*Lactuca sativa*)				- Leaf Se contents up to 15 µg g^-1^ DW in response to 5.2 μmol L^−1^	30-fold change	Francini et al., 2023
11	Spinach (*Spinacia oleracea*)				- Leaf Se contents up to 15 µg g^-1^ DW in response to 5.2 μmol L^−1^	30-fold change	Francini et al., 2023
12	Wheat (*Triticum aestivum* L.)	Sodium selenite and sodium selenate	0, 10 µM and mixture of both	Root zone/Hydroponics	- Grain Se contents were 150 mg kg^-1^ in response to selenite treatment	300-fold change	Subirana et al., 2023
13	Psyllium (*Plantago ovata*)	sodium selenate	0, 10, 50, 100 and 500 µM	Agar medium/Root zone	- Highest accumulation of 457.65 µg g^-1^ FW at 500 µM Se treatment	~250-fold change	Dey and Raychaudhuri, 2024

### 3.2 Foliar application of Se on plants

Foliar application of Se is an effective approach for directly delivering Se to leaves. Unlike Se immobilization in soils, foliar Se application promotes its uptake and transport in leaves. This approach utilizes a low concentration of Se compounds and is safe, convenient, and cost-effective, ensuring optimized and precise Se delivery to plants (de Lima et al., 2023). For instance, the foliar application of Na_2_SeO_3_ increased the Se fraction in strawberries, which was further reduced to Se^2-^ and assimilated into organic forms (Lin et al., 2024). Several other studies have reported the success of foliar Se sprays in enhancing the Se content of plants. For instance, foliar spray of Se at a concentration of 200 mg L^-1^ at different fruit development stages substantially enhanced the Se fraction in leaves and citrus fruits compared with soil application (Wang et al., 2024). Rice Se levels were enhanced–5–6 times with a foliar spray of selenite (Lidon et al., 2019). Similarly, foliar application of Na_2_SeO_3_ enhanced Se enrichment in persimmons (Yan et al., 2021), jujubes (Wang et al., 2020a), and strawberries (Huang et al., 2023).

Two key enzymes, O-acetyl serine (OAS) and cysteine synthase, convert Se^2-^ into SeCys, which can be further methylated into methyl-SeCys (by selenocysteine methyltransferase) or SeMet by selenocysteine lyase (Adebayo et al., 2020). Similarly, organic Se is the dominant assimilated form of Se in fruit (Sarwar et al., 2020; Wang et al., 2024). It has also been reported that Se-amino acids can replace S-containing amino acids in proteins (Zagrodzki et al., 2023). However, this depends on several factors, including Se concentration, Se compound application, mode of application, and plant developmental stages (Schiavon et al., 2017). Recently, we reported that foliar spray of Na_2_SeO_3_ at concentrations up to 200 mg L^-1^ improved fruit quality in *Citrus reticulata* and enhanced Se content in fruit by 3.6 folds (Wang et al., 2024). At the field scale, the repeated application (four applications per year) of Na_2_SeO_4_ in combination with CaCl_2_ enhanced cherry fruit Se content up to 12 folds (Wójcik, 2024). So far, Se biofortification has been successfully achieved in fruits, vegetables, and cereal crops through foliar application, as summarized in **Table 3**.

**Table 3:** Biofortification of Se in horticultural crops by foliar application – recent reports.

Sr. No.	Plant species/part	Se compound applied	Se concentration applied	Mode of application	Physiological effects/ Se biofortification	Fold change (compared to control)	References
1	Broccoli (*Brassica oleracea* var. *italica*)	Na_2_SeO_4_ Na_2_SeO_3_	0, 1 and 2 mM	Foliar spray	- 27.2 µg g^-1^ DW Se in leaves by 2 mM selenate - 40.4 µg g^-1^ DW Se by 2 mM selenite application	100-fold 149-fold	Buttarelli et al., 2025
2	Spinach (*Spinacia oleracea* L.)	Na_2_SeO_4_ combined with KI	1, 2.5 and 5 mg L^−1^ combined with iodine (1, 2.5 and 5 mg L^−1^)	Foliar spray	- Maximum improvement in plant yield at 2.5 mg L^−1^ Se application - 63.5 ng/g Se in response to 5 mg L^−1^ application	2.43-fold	Eslamiparvar et al., 2025
3	Alfalfa (*Medicago sativa* L.)	Nano-selenium (diameter 65–106 nm)	0, 30, 50, 100, 150 and 250 mg L^-1^	Foliar spray	- Increase in SeMet and SeMeCys concentration - 16 mg kg^-1^ Se in leaf - 10 mg kg^-1^ Se in stem - 1.5 mg kg^-1^ Se in root	80-fold 50-fold 7.5-fold	Sun et al., 2025
4	Citrus (*Citrus reticulata*)	Na_2_SeO_3_	0, 25, 50, 100 and 200 mg L^-1^	Foliar spray	- Fruit weight and quality were improved - 12.26 ng g^-1^ Se fruit - 115.9 ng g^-1^ Se in leaf	3.63-fold 2.05-fold	Wang et al., 2024
5	Onion (*Allium cepa* L.)	Na_2_SeO_4_	0, 10, 25, 50, 100 and 200 g ha^-1^	Foliar spray	- Increase in onion yield up to 99 kg ha^-1^ - 0.706 mg kg^-1^ Se in bulb	70.6-fold	Machado et al., 2024
6	Chard (*Beta vulgaris* var. *cicla*)	2-iminoselenazolidin-4-ones (ISeA)	0 and 2 mg L^-1^	Foliar spray	- Fresh biomass increased - 35.7 mg kg^-1^ Se in leaves	36-fold	Semenova et al., 2024
7	Komatsuna (*Brassica rapa* var. *perviridis*)	2-iminoselenazolidin-4-ones (ISeA)	0 and 2 mg L^-1^	Foliar spray	- Growth enhancement - 31.1 mg kg^-1^ Se in leaves	31-fold	Semenova et al., 2024
8	Sweet cherry (*Prunus avium*)	Na_2_SeO_4_ in combination with CaCl_2_	15 g ha^−1^ spray^−1^ (four times application per season)	Foliar spray	- Leaf Se up to 1.258 mg kg^-1^ (two-year average) - Fruit Se up to 3.65 µg 100 g^-1^ FW (two-year average) - Combined application with CaCl_2_ further enhanced Se biofortification - No leaf or fruit injury by Se application	29.2-fold 12.1-fold	Wójcik, 2024
9	Lettuce (*Lactuca sativa* L.)	Na_2_SeO_4_	0 and 25 mg L^-1^	Foliar spray	- Seed production was enhanced by 20–41% due to Se application -Prominent biofortification in lettuce up to 50 times - Se treatment in combination with garlic extracts further enhanced Se biofortification	40–50 folds	Golubkina et al., 2024a
10	Wheat (*Triticum aestivum* L.)	Selenate and selenite nanocarriers/Liposomes	100, 500 and 1000 μM	Foliar spray at 5^th^ and 7^th^ week leaf stage	- The shoot Se concentration of 3.8 µg g^-1^ and 15 µg g^-1^ DW after single and double application of 1000 µM/liposome nanocarrier	7.6-folds after single application 30-folds by twice application	Viltres-Portales et al., 2024
11	Rice (*Oryza sativa* L.)	SeNPs and Se (IV)	0 and 0.5 mg L^-1^	Foliar spray at flowering stage	- SeNPs enhanced Se in husk (0.418 µg g^-1^ DW) & grain (0.462 µg g^-1^ DW) - Se(IV) enhanced Se in husk (0.627 µg g^-1^ DW) & grain (0.701µg g^-1^ DW)	2.47-fold in husk and 2.73-fold in grain 3.71-fold in husk and 4.14-fold in grain	Freire et al., 2024
12	Soybean (*Glycine max*)	Sodium selenate	0, 15, 40 or 100 g Se ha^-1^	Foliar spray at flowering stage	- The Se concentration in seeds was up to 15 mg kg^-1^ highest treatment - In leaf, the Se was 3.5 mg kg^-1^ at highest treatment	150-folds 35-folds	Mrština et al., 2024
13	Basil (*Ocimum basilicum*)	Sodium selenate	0 and 5 μM	Foliar spray	- The leaf Se was 30.2 mg kg^-1^ DW	36.4-folds	Profico et al., 2025
14	Mint (*Mentha* spp.)	Sodium selenate	0 and 5 μM	Foliar spray	- The leaf Se was 27.6 mg kg^-1^ DW	29.3-folds	Profico et al., 2025
15	Maize (*Zea mays* L.)	ZnSO_4_·7H_2_O, FeSO_4_·7H_2_O and Na_2_SeO_3_ cocktail	0 and 2000 ppm	Foliar spray	- Enhancement in the Se in shoot (683.9 µg kg^-1^), straw (1016.0 µg kg^-1^) and grain (413.4 µg kg^-1^)	13.1-fold (shoot) 11.0-fold (straw) 19.4-fold (grain)	Xue et al., 2023

## 4 Se-uptake, transport, and assimilation pathways and genes involved therein

### 4.1 Selenium uptake

Plant roots exhibit different uptake pathways for selenate (SeO_4_^2-^) and selenite (SeO_3_^2-^), which vary among Se non-accumulator and hyperaccumulator species (Lima et al., 2018). It was initially believed that selenite enters plant roots by passive diffusion, but later studies confirmed the active root uptake of Se species via low- and high-affinity transporters (Shrift and Ulrich, 1969; Zhang et al., 2003; Li et al., 2008). Moreover, the root uptake capacity of different Se species varies depending on pH, with HSeO_3_^−^ being the dominant form taken up by tomato and maize plants (Zhao et al., 2010; Zhang et al., 2010; Wang et al., 2019). Interestingly, the uptake of both selenate and selenite follows different uptake pathways and transporters in the root cell plasma membrane, and both S and P are two macronutrients affecting Se uptake by roots, which have been discussed in detail.

#### 4.1.1 Selenate [Se(VI)] uptake by sulphate transporters

As mentioned earlier, selenate [SeO_4_^2-^; Se(VI)] dominates alkaline soils because of its structural and chemical similarities to S and is primarily taken up by roots via sulfate transporters (Anderson, 1993; Sors et al., 2005; White, 2016). In general, sulfate transporters belong to four gene families: SULTR1, SULTR2, SULTR3, and SULTR4, which primarily regulate S uptake, transport, and regulation within plants (El Mehdawi et al., 2018).

Among these, SULTR1 transporters are high-affinity S-transporters (H^+^/sulfate symporters) localized inside root cells (Sors et al., 2005; Shinmachi et al., 2010). In *Arabidopsis*, *AtSULTR1;2* is highly expressed in the root tips, lateral roots, and root cortex (Shibagaki et al., 2002). Both SULTR1;1 and SULTR1;2 mediate root S uptake in plants. Interestingly, *AtSULTR1;2* knockout mutants exhibit reduced S and Se uptake in roots, suggesting their involvement in Se uptake (Ohno et al., 2012). Similarly, elevated expression of *SULTR1* genes has also been found in Se-hyperaccumulator plant species (Cabannes et al., 2011; El Mehdawi et al., 2018). Elevated expression of *SULTR1;1* is consistent with increased selenate uptake in wheat and lettuce (Ramos et al., 2011; Boldrin et al., 2016). Similarly, both AtSULTR1;1 and AtSULTR1;2 catalyze root Se uptake in *Arabidopsis* under high and normal Se conditions (Rouached et al., 2008). These reports suggest a substantial involvement of SULTR1 transporters in root selenate uptake from the soil solution. In contrast, the SULTR2 gene family is involved in S and Se xylem loading and transport to aboveground plant parts. The Se hyperaccumulator plant species often exhibit high *SULTR2;1* expression (Cabannes et al., 2011; Wang et al., 2018). Moreover, increased *SULTR2;1* expression has been reported in broccoli, rice, and tea plants in response to exogenous selenate application, confirming its role in Se transport (Zhang et al., 2020a; Ren et al., 2022; Yang et al., 2022). Additionally, SULTR3 transporters are involved in chloroplast S uptake, whereas SULTR4 transporters are involved in S efflux from the vacuole.

As no dedicated Se transporters have been identified in plants, selenate uptake in plants primarily follows the S-transport pathway. This suggests that root Se uptake occurs via SULTR1, followed by xylem loading via SULTR2, and then transport to the chloroplast via SULTR3 for possible reduction to selenite and incorporation into organic compounds. Consistent with this assumption, selenate treatment increases the expression of *SULTR3;1*, *SULTR3;5*, and *SULTR4;1* in cabbage, wheat, and broccoli, respectively (Boldrin et al., 2016; Wu et al., 2022; Yang et al., 2022). Se/S homeostasis via vacuolar sequestration may involve the SULTR4. The ATP-Binding Cassette (ABC) transporters and MOTI (which functions in molybdate/sulfate transport across the plasma membrane) may also be involved in root uptake of Se; however, this has not yet been reported.

#### 4.1.2 Selenite [Se(IV)] uptake by phosphate transporters

Initially, it was believed that selenite (SeO_3_^2-^) uptake by roots was a passive process, but later studies confirmed its active uptake at rates comparable to or even higher (Hopper and Parker, 1999; Li et al., 2008). Phosphate application has also been found to suppress SeO_3_^2-^ uptake (Hopper and Parker, 1999). Similarly, increasing P supply inhibits SeO_3_^2-^ root uptake in rice, whereas P starvation promotes its uptake (Li et al., 2019; Wang et al., 2019). Studies have supported the notion that SeO_3_^2-^ uptake is primarily mediated by phosphate transporters (PHTs) (Li et al., 2008; Zhang et al., 2014; Song et al., 2017). In general, phosphate transporters can be classified into four gene families: PHT1, PHT2, PHT3, and PHT4 based on sequence, conserved domains, and functions (Okumura et al., 1998; Rausch and Bucher, 2002; Zhang et al., 2016; Sun et al., 2017). The PHT1 transporters are localized in the plasma membrane, whereas PHT2 and PHT3 are localized in the plastid inner envelope and mitochondrial membrane, respectively (Knappe et al., 2003; Młodzińska and Zboińska, 2016). Furthermore, PHT4 is located within plastids and the secretory system (Guo et al., 2008).

In tea plants, Se-mediated regulation of *CsPHT1* has been reported, suggesting its role in Se uptake (Cao et al., 2021). Similarly, *OsPT2* and *OsPT8* contribute to the uptake of selenite in rice. The PHT1 subfamily encodes an H^+^ symporter involved in Pi uptake in *Arabidopsis thaliana* (Młodzińska and Zboińska, 2016). Additionally, it has been proposed that a conformational change in the phosphate-H^+^ symporter contributes to SeO_3_^2 −^ anion uptake (Song et al., 2017). In contrast, AtPHT2;1 is involved in Pi translocation and allocation to leaves (Daram et al., 1999; Versaw and Harrison, 2002). However, OsPT2 is involved in selenite uptake (Zhang et al., 2014). Interestingly, *CsPHT* gene expression was upregulated in response to low, medium, and high Se treatments and positively correlated with Se uptake (Cao et al., 2021). It is pertinent to mention that the PHT3 subfamily in *Arabidopsis* is expressed in the stems, leaves, and flowers (Poirier and Bucher, 2002). As *the PHT3* gene encodes mitochondrial Pi transporters, it suggests a major role in Se uptake and reduction. Taken together, these results suggest that PHT2 and PHT3 are involved in Se translocation to leaves and its subsequent allocation to plastids, where it is further reduced to Se-organic compounds. In addition, the expression of *AtPHT4* genes has been reported in the roots and leaves of *Arabidopsis* and *AtPHT4;6* regulates Pi transport from the Golgi to the cytosol (Guo et al., 2008). Additionally, PHT4;2 contributes to Pi transport in isolated root plastids and was also identified as a plastidic Pi translocator, pPT, whose expression is mainly confined to roots during vegetative development (Irigoyen et al., 2011). Recently, upregulation of *CsPHT4* gene expression in response to Se application has been linked to endomembrane function (Cao et al., 2021). In contrast, we propose that the function of PHT4 is in tandem with that of PHT2, and therefore, it contributes to the synthesis of organic Se compounds inside root plastids. Therefore, root plastids import ATP from the cytosol (Irigoyen et al., 2011), which likely aids in SeO_3_^2-^ reduction.

Little is known about how other phosphate transporters, including PHT5, SPX, and PHO1 regulate selenite uptake and metabolism in plants. It is pertinent to mention here that PHT5 is a vacuolar phosphate transporter1 localized in the tonoplast involved in Pi homeostasis, whereas PHO1 is involved in xylem Pi loading and is present in root vasculature, and SPX is reported to regulate Pi signaling (Wang et al., 2015; Liu et al., 2016). Additionally, PHO1 is localized in the plasma membrane and Golgi apparatus, and plays a role in root-shoot xylem transport (Arpat et al., 2012; Lopez-Arredondo et al., 2014). Therefore, if we draw parallels by following the path of Pi, it could be speculated that PHO1 might regulate selenite xylem loading and its subsequent translocation to the shoot, whereas PHT5 may be involved in cellular Se homeostasis. Depending on the soil pH, selenite exists as either H_2_SeO_3_ (at pH 3), HSeO_3_^−^ (at pH 5), or SeO_3_^2−^ (at pH 7) and these Se-species are differentially taken up different phosphate transporters. As mentioned earlier, phosphate transporters and anion channels contribute to the uptake of HSeO_3_^−^ and SeO_3_^2−^ in plants which exist at pH 5 and pH 7 respectively (Zhang et al., 2014; Zhao et al., 2024). However, the H_2_SeO_3_ uptake is mediated by the silicon transporters (Zhao et al., 2010; Wang et al., 2019). Therefore, further studies are required to validate these assumptions to improve our understanding of the comparative regulation of P and Se within plant cells.

#### 4.1.3 Involvement of nitrate, silicon, and aluminum transporters

The role of these transporters in Se uptake is not properly understood, as only a few studies have reported the interactions of NO_3_^2-^, Si, and Al transporters in Se uptake. Wang et al. (2022b) reported the transport of SeMet by NRT1.1B from leaves to seeds. Aquaporin and Si influx transporters, OsNIP2;1, have been reported to enhance the uptake of SeO_3_^2-^ in rice and yeast (Zhao et al., 2010; Xu et al., 2023). However, Zhao et al. (2010) showed that increasing the Si concentration in the solution affects Se uptake in rice roots. Moreover, the selenite uptake greatly enhanced at acidic pH up till 5.5 and the authors concluded that the Si- transporter (OsNIP2;1) is permeable to SeO_3_^2-^ (Zhao et al., 2010). In another study, Wang et al. (2019) also reported that root SeO_3_^2-^ in tomatoes remained unaffected by Si addition at a pH above 5. However, their study provided evidence that H_2_SeO_3_ can be taken up by the roots by Si transporters in tomatoes. Other candidates include members of the Aluminum-activated Malate Transporter (ALMT) family. AtALMT12 was found to be involved in root SeO_4_^2-^ xylem loading, potentially involving the SULTR2 and SULTR3 transporters (Gigolashvili and Kopriva, 2014). In short, these reports suggest a very limited or indirect role for Si influx transporters in root Se uptake.

### 4.2 Selenium accumulation and transport

Inside the plant cells of roots and shoots, the majority of Se (60-75%) is adsorbed onto the cell wall (Ding et al., 2015). Some Se remains in the cytosol in its soluble form, while others are transported to organelles (Wang et al., 2020c). In addition, the vacuoles of the leaf mesophyll cells and vasculature also serve as a major sequestration point for Se (Ximénez-Embún et al., 2004; Wang et al., 2015) and AtSULTR4;1 and AtSULTR4;2 possibly mediate Se efflux from vacuoles (Kataoka et al., 2004; Gigolashvili and Kopriva, 2014). Selenium hyperaccumulators typically exhibit elevated expression of genes encoding both these transporters as well as in response to Se supplementation, thereby suggesting a significant role in Se homeostasis (Both et al., 2020; Wang et al., 2022d; Yang et al., 2024; Zhao et al., 2025).

The translocation mechanisms for both selenate and selenite are also substantially different depending on the Se compound applied. Selenate is the dominant Se species in the vasculature (Li et al., 2008). Earlier Zayed et al. (1998) reported that selenate supplementation caused a Se _shoot_/_root_ ratio ranging between 1.4-17.2, whereas selenite caused a ratio less than 0.5. Similarly, rice plants treated with selenite exhibited a translocation factor of 0.21, indicating limited transport to the shoots (Wang et al., 2020c). This clearly indicates that selenate is more actively translocated to the shoot than selenite. In contrast, selenite transport was very low, further indicating its assimilation into Se organic compounds in roots (Huang et al., 2017). Therefore, selenate follows a symplast pathway from root to shoot, and SULTR transporters are primarily involved in leaf selenate uptake (White et al., 2004; Li et al., 2008; Takahashi et al., 2011; Gigolashvili and Kopriva, 2014). However, selenoamino acids synthesized from selenite reduction in roots are directly translocated to shoots via xylem tissue (Li et al., 2008; Guignardi and Schiavon, 2017). Moreover, the phloem redistribution of both selenate and organo-Se compounds has been reported (Carey et al., 2012), which suggests an active source-to-sink relationship and Se homeostasis. Nonetheless, AtSULTR1;3, AtSULTR2;2, and amino acid transporters facilitate Se transport through the phloem (Takahashi et al., 2000; Yoshimoto et al., 2003; Tegeder, 2014; Boldrin et al., 2016; Gao et al., 2024).

Apart from this, plant roots can uptake various organic Se forms, particularly selenoamino acids (SeMet, SeCys and MeSeCys) and selenoproteins (released from decaying organic matter). In fact, the uptake of organic forms of Se in both wheat and canola was way higher than inorganic forms. A 300-minute exposure of durum wheat roots to 5 µM Se treatments resulted in selenate (5.9 µg Se g^-1^ DW), selenite (15 µg Se g^-1^ DW), SeCys (60 µg Se g^-1^ DW) and SeMet (350 µg Se g^-1^ DW) respectively (Kikkert and Berkelaar, 2013). Likewise in canola, the root uptake of selenate (45 µg Se g^-1^ DW), selenite (68 µg Se g^-1^ DW), SeCys (109 µg Se g^-1^ DW) and the SeMet was around 1800 µg Se g^-1^ DW (Kikkert and Berkelaar, 2013). In algae, the SeMet uptake inhibited methionine suggesting the involvement of amino acid transporters in Se-amino acids (Sandholm et al., 1973). Therefore, selenoamino acids may be taken up by H^+^-coupled amino acid transporters via both symplastic and apoplastic routes. Furthermore, the Amino Acid Permeases (AAPs) and Lysine-Histidine like Transporters (LHTs) might be involved in Se-amino acid uptake and its transport through plant vasculature (Guo et al., 2021).

### 4.3 Selenium assimilation

Generally, it is believed that Se assimilation primarily occurs in roots, and upon its uptake, Se can be incorporated into Se-amino acids (SeCys), SeMet, and MeSeCys) through the S-metabolic pathway (Whanger, 2002; Sors et al., 2005; Rayman et al., 2008). Multiple studies have confirmed the presence of seleno-amino acids, such as SeCys, MeSeCys, and SeMet, in roots when compared to shoots (Huang et al., 2017; Hu et al., 2018).

In contrast, Li et al. (2008) showed that selenate is actively transported to wheat shoots (without being incorporated into amino acids). In contrast, selenite was rapidly incorporated into Se-amino acids by the roots within 3–4 minutes of supplementation. Similarly, selenite-treated rice roots exhibited SeMet as the dominant Se species (Yu et al., 2019). This clearly shows the preference of the roots for the reduced Se form (selenite) over the oxidized form (selenate), which can be explained by several factors. It has also been reported that the SeO_4_^2-^ to SeO_3_^2-^ reduction is the rate-limiting step in plant Se metabolism in non-hyperaccumulators (Sors et al., 2005). First, plant tolerance to Se compounds varies, and organo-Se compounds are believed to be safer than inorganic forms (Thiry et al., 2012). Therefore, it is safer to convert available Se to Se-amino acids within the roots. In addition, the reduction of inorganic Se into organo-Se requires reducing equivalents, and this process exclusively occurs within plastids. The plastids in the roots have a very limited supply of reducing power; therefore, they prefer selenite over selenate. Selenoamino acids are primarily synthesized inside the leaf chloroplasts (Terry et al., 2000; White, 2018b; Trippe III and Pilon-Smits, 2021). In the leaf, selenate is transported to plastids via the homologs of AtSULTR3;1 located at chloroplast membranes, where it is reduced and incorporated into Se-amino acids through S-metabolism. Additionally, the seleno-amino acids taken up by the roots be directly incorporated into the proteins. The assimilation of SeMet might undergo activation by methionyl-tRNA synthetase which results in the formation of selenomethionyl-tRNA which then incorporated into the newly synthesized proteins by ribosomes. Likewise, the SeCys can be incorporated into the proteins substituting cysteine (which can also lead to disruption of disulfide bonds).

**Figure 1:**
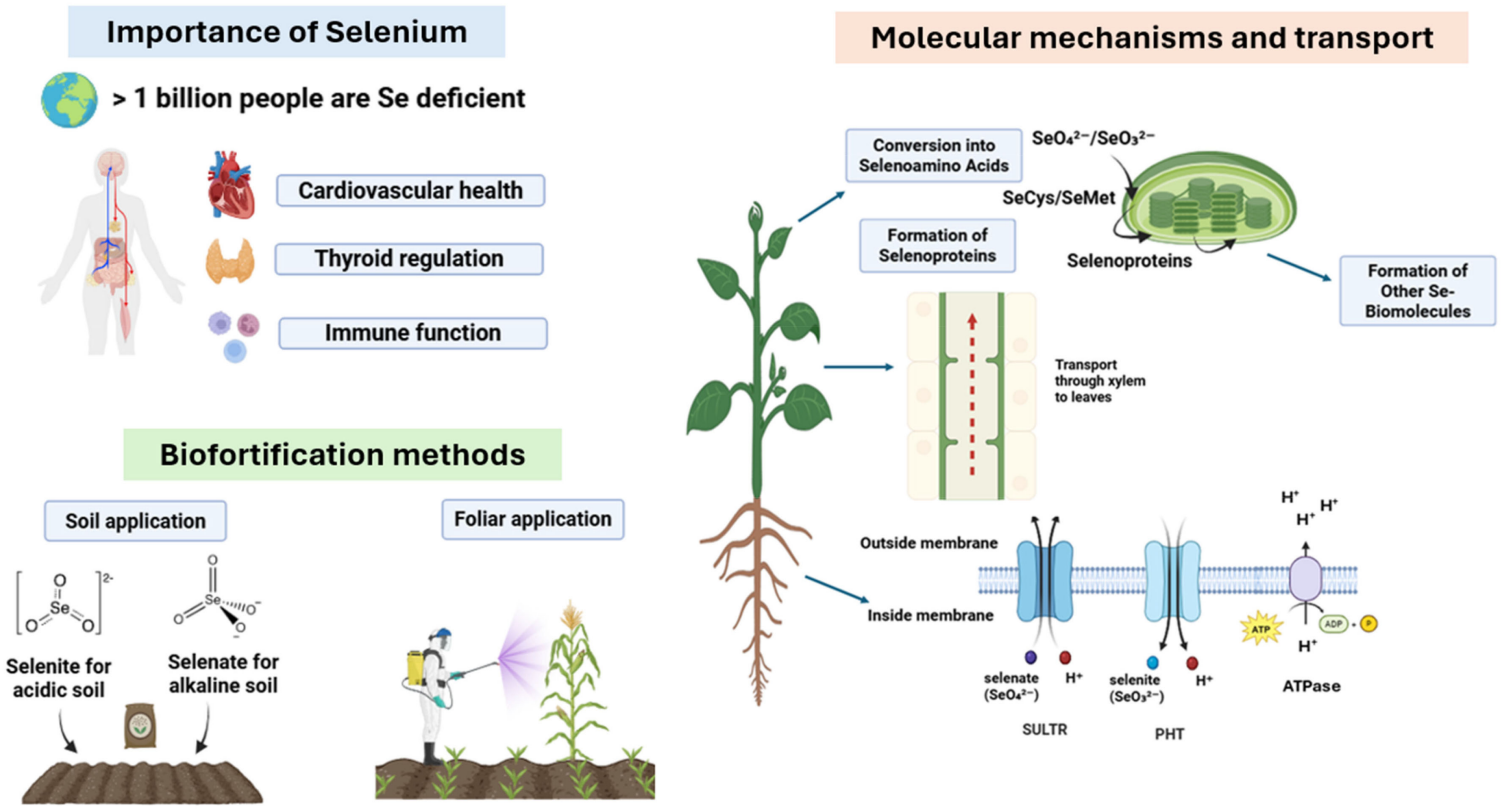
Schematic diagram showing the importance and methods for enhancing Se biofortification in crops alongside molecular mechanisms involved therein.

Selenium hyperaccumulators typically exhibit elevated expression of genes involved in S-metabolism, which becomes very high in Se hyperaccumulators (Van Hoewyk et al., 2005, 2008; Schiavon et al., 2015). First, adenosine triphosphate sulfurylase (APS) converts selenate into adenosine 5′- phosphoselenate, also called APSe (Bohrer et al., 2015). Later, selenoglutathione and selenolipids were formed from APSe via analogous S pathways (Duncan et al., 2017). Selenite can also be synthesized by adenosine 5′- phosphosulfate reductase (APR), which is a rate-limiting step that uses glutathione as an electron donor (Pilon-Smits et al., 1999; Wang et al., 2018). Furthermore, selenite reduction can be enzymatically (by sulfite reductase) or non-enzymatically (glutathione oxidoreductase), facilitating its incorporation into Se-amino acids (González-Morales et al., 2017; Van Hoewyk and Çakir, 2017; Zhong et al., 2024). Once synthesized, selenoamino acids are incorporated into selenoproteins (**Figure 1**).

### 4.4 Seleno-biomolecules

Both Se and S have different ionization energies and atomic/ionic radii, which influence protein structure and folding properties. The radius of Se^2+^ is 0.5A°, whereas that of S^2+^ is 0.37 A° (Terry et al., 2000). In contrast, S and Se share a similar primary metabolism, and accumulation of Se in tissues can replace S-rich metabolites, thereby mediating a wide range of plant physiological responses (reviewed by White, 2018a). Se accumulation in albumin, globulins, and prolamin has been reported previously (Liu et al., 2011; Nwachukwu and Aluko, 2018; Zhang et al., 2021). In addition, selenoproteins exhibit better redox activity and utilize SeCys as a catalytic residue, which provides greater nucleophilicity, reactivity, and resistance to permanent oxidation (Reich and Hondal, 2016; Schomburg and Arnér, 2017). In addition, some studies have reported that Se bioaccumulation promotes SOD synthesis, thereby assisting antioxidant capacity (Georgiadou et al., 2018). Alternatively, the exogenous application of Se as a foliar spray enhances gene expression related to antioxidant defense pathways (Lanza and Reis, 2021).

Apart from selenopeptides and antioxidants, various metabolites are derived from SeMet and SeCys (Reviewed by White, 2018). These include selenoglutathione (Crawford et al., 2000; Pivato et al., 2014), S-allyl-L-cysteine sulfoxide (González-Morales et al., 2017), and selenoglucosinolates (Matich et al., 2012; Ouerdane et al., 2013; Matich et al., 2015). Moreover, foliar application of Se has been reported to enhance quality traits in citrus (Wang et al., 2024), potatoes (Ibrahim and Ibrahim, 2016), sapotas (Lalithya et al., 2014), and pomegranates (Zahedi et al., 2019). Studies have also revealed Se-mediated enhancement in the enzymatic activity of fructose 1,6-bisphosphatase, resulting in the enhancement of total soluble sugars in citrus plants (Owusu-Sekyere et al., 2013). Interestingly, selenosugars and selenopolysaccharides have been reported in cereal crops (Aureli et al., 2012; Qi et al., 2024). In addition, exogenous application of Se can also influence ascorbic acid content in citrus fruits (Ibrahim and Al-Wasfy, 2014; Zhan et al., 2021; Wen et al., 2021). Finally, selenoflavonoids have been isolated from various plants. These include the isolation of Se-containing 5,7-dihydroxychromone (Se-DHC) and flavones from Se-enriched green tea (Fan et al., 2022). Se assimilation into biomolecules in plants follows a very diverse route and is actively involved in plant growth, resilience, and survival. Lastly, several studies have reported that exogenous Se led to an increase in the fruit quality characteristics and protein contents. Some of the studies have been summarized in **Table 4**.

**Table 4:** Protein quality improvement by Se biofortification in horticultural crops.

Sr. No.	Horticultural crop	Se form and application	Se concentration applied	Physiological effects with an emphasis on protein quality	References
1.	Tomato (*Solanum lycopersicum*)	Foliar application of sodium selenate and Se nanoparticles	10 mg L^-1^	- Se enrichment influenced both primary and secondary metabolites - Changes in fruit biochemical composition including protein-related quality parameters	Shiriaev et al., 2023
2.	Cucumber (*Cucumis sativus*)	Foliar application of sodium selenate	0, 5, and 10 mg L^-1^	- Se-biofortified plants exhibited improvements in proline, total soluble sugars and proteins - Phenol, flavonoids, and antioxidant enzymes were also enhanced under salinity stress	Amerian et al., 2024
3.	Potato (*Solanum tuberosum*)	Nano-Se fertilizer having particle size of 50–78 nm	0 and 1500 mg L^-1^	- Increase in crude protein and vitamin C - Tuber Se content was significantly enhanced along with potato yield and tuber quality - Up-regulation of antioxidant enzyme activities of GSH, POD, PPO, SOD and PAL	Liu et al., 2025
4.	African eggplant (*Solanum macrocarpon* L.)	Se-fortified fertilizer in the form of sodium selenite	0, and 1 g kg^-1^	- A 20% increase in Se concentration from 5.91 ± 0.02 mg 100 g^-1^ to 101.01 ± 0.05 mg 100 g^-1^ - Significant improvement in the proximate composition of fruit including crude protein	Ogunsuyi et al., 2025
5.	Citrus (*Citrus reticulata*)	Soil, foliar and combined applications (Soil + Foliar)	0, 25, 50, 75, 100 and 200 mg L^-1^	- Fruit Se contents increased during all stages of development - Improvement in fruit quality traits including protein contents	Wang et al., 2024
6.	Onion (*Allium cepa*)	Soil and foliar application of sodium selentae	0, 10, 25, 50, 100 and 200 g ha^-1^	- Increase in Se content in the bulb and its dry biomass - improvements in bulb quality traits	Machado et al., 2024
7.	Broccoli(*Brassica oleracea var. italica*)	Foliar spray of selenate and selenite	0, 1 and 2 mM	- Increase in total soluble proteins by +2.2 folds - Se-biofortification also enhanced crop mineral nutrition	Buttarelli et al., 2025
8.	Perennial wall rocket (*Diplotaxis tenuifolia* L. -DC)	Foliar spray of sodium selenate	0, 50 mg Se L^−1^ (0.26 mM)	- Prominent improvements in the protein (%) at three crop cycles - Increase in Se contents as well as Ca, Mg, K, P, Fe, Cu, and Zn	Tallarita et al., 2025
9.	Pakchoi (*Brassica rapa* subsp. *chinensis*), Lettuce (*Lactuca sativa*) and Beans (*Phaseolus* *vulgaris*)	Soil application of sodium selenate and selenite	0, 0.5, 1, 3 and 5 mg kg^-1^	- Improvements in crop quality traits - Enhancement in yield characteristics - Both shoot and root Se concentrations were enhanced - Crop quality traits improved	Li et al., 2025
10.	Tomato (*Solanum lycopersicum*)	Foliar spray of sodium selenite at fruit expansion stage	0 and 25 μM	- Application of Se increased the soluble protein concentration - Increase in yield and quality of tomatoes	Hu et al., 2023
11.	Lettuce (*Lactuca sativa*)	Foliar application sodium selenite	0, 0.5, 1, 1.5 and 2 mg L^-1^	- Significant increase in the soluble proteins at low concentrations	Cheng et al., 2024
12.	Carrot (*Daucus carota* subsp. *sativus*)	Root zone application of Se-NPs	10 mg L^-1^	- Se NPs individually or under stress conditions improved the soluble protein content	El-Batal et al., 2023
13.	Mung bean (*Vigna radiata*)	Foliar spray of sodium selenite	CK, 15, 30, and 45 g ha^−1^	- 30g ha^-1^ Se concentration significantly increased the contents of protein in the two mung bean varieties	Wang et al., 2022a
14.	Cowpea (*Vigna unguiculata*)	Seed pre-treatment with sodium selenate and Se-NPs	0.0, 6.25, 12.5, 25, 50 and 100 μM	- Enhanced total soluble proteins were recorded cowpea seedlings at lower concentrations	Zeid et al., 2019
15.	Soybean (*Glycine max*)	Foliar application of Se-NPs	5, 10, and 20 mg L-¹	- Positive relationship between SeNPs application and soybean seed protein content compared to control was observed	Ssemalawa et al., 2025
16.	Tomato (*Solanum lycopersicum* L.)	Root zone application of Se-NPs	0, 100, 300 mg L^-1^	- SeNPs improved the protein contents of the tomato plants under Cd-stress	Ahmad et al., 2024
17.	Potato (*Solanum tuberosum* L.)	Se nanopowder applied as seed pre-treatment	400 g ha^-1^	- Nano-Se application led to an increase in the protein content	Chernikova et al., 2019

## 5 Selenium toxicity in plants

Since Se is not essential for most plants and can be toxic at high levels and inhibit plant growth, development and physiological processes, leading to cause symptoms such as chlorosis, necrosis, reduced protein synthesis and stunted growth (Schiavon et al., 2017). Exposure to high Se concentration also caused reduced biomass, lower seed germination rates and decreased pollen production in *Brassica juncea* (Kushwaha et al., 2022). The Se toxicity is also known as selenosis and primarily arises forming seleno-proteins and the induction of oxidative stress (Hasanuzzaman et al., 2020). Toxic concentration of Se in plant tissues is typically above 5 mg kg^-1^, however the Se tolerance among different crop plants varies widely. For example, rice and wheat showed toxicity symptoms at 2 and 4.9 mg kg^-1^ DW Se concentration respectively; whereas Dutch clover tolerated 330 mg kg^-1^ DW Se without toxic symptoms (Kolbert et al., 2019). Not only this, the severity of Se toxicity also varies depending on the plant species, its age and the level of Se exposure. Younger plants are generally more susceptible to Se toxicity compared to mature plants and selenite (SeO_3_^2-^) is known to be more harmful than selenate (SeO_4_^2-^) (Hasanuzzaman et al., 2020). Basically, Se toxicity in plants can be due to selenoproteins-induced toxicity or Se-induced oxidative stress (Ali et al., 2021). Misfolded selenoproteins are produced when SeCys or SeMet are mistakenly incorporated into protein chains in place of cysteine (Cys) or methionine (Met) which affects tertiary structure of proteins. Not only this, the protein stability, enzyme activity, redox potential and its enzyme kinetics are affected leading to toxicity (Reviewed by Gupta and Gupta, 2017). Furthermore, the replacement of Cys with SeCys at the metal binding site increase bond due to larger atomic radii of Se potentially affecting its interaction with the co-factors (Van Hoewyk, 2013). Moreover, Se can act as a pro-oxidant, thereby stimulating over-production of ROS leading to Se-induced phytotoxicity. For instance, the reaction between selenite (SeO_3_^2-^) and glutathione (GSH) generates superoxide radicals (O_2_^-^), resulting in the subsequent accumulation of hydrogen peroxide (H_2_O_2_). As a result, excessive Se depletes GSH levels and reduces its activity, thereby promoting further ROS generation (Hasanuzzaman et al., 2020). Lastly, Se accumulation can also interfere nutrient uptake. It has been reported that excessive Se negatively affects N-metabolism through inhibition of nitrate reductase activity by affecting synthesis of molybdopterin, which serve as cofactor for nitrate reductase and essentially required for nitrogen assimilation. This in turn disturbs N-metabolism and synthesis of amino acids, proteins, nucleotides and vital secondary metabolites affecting a wide range of developmental processes in plants (Gouveia et al., 2020).

## 6 Conclusions and future perspectives

**Figure 2:**
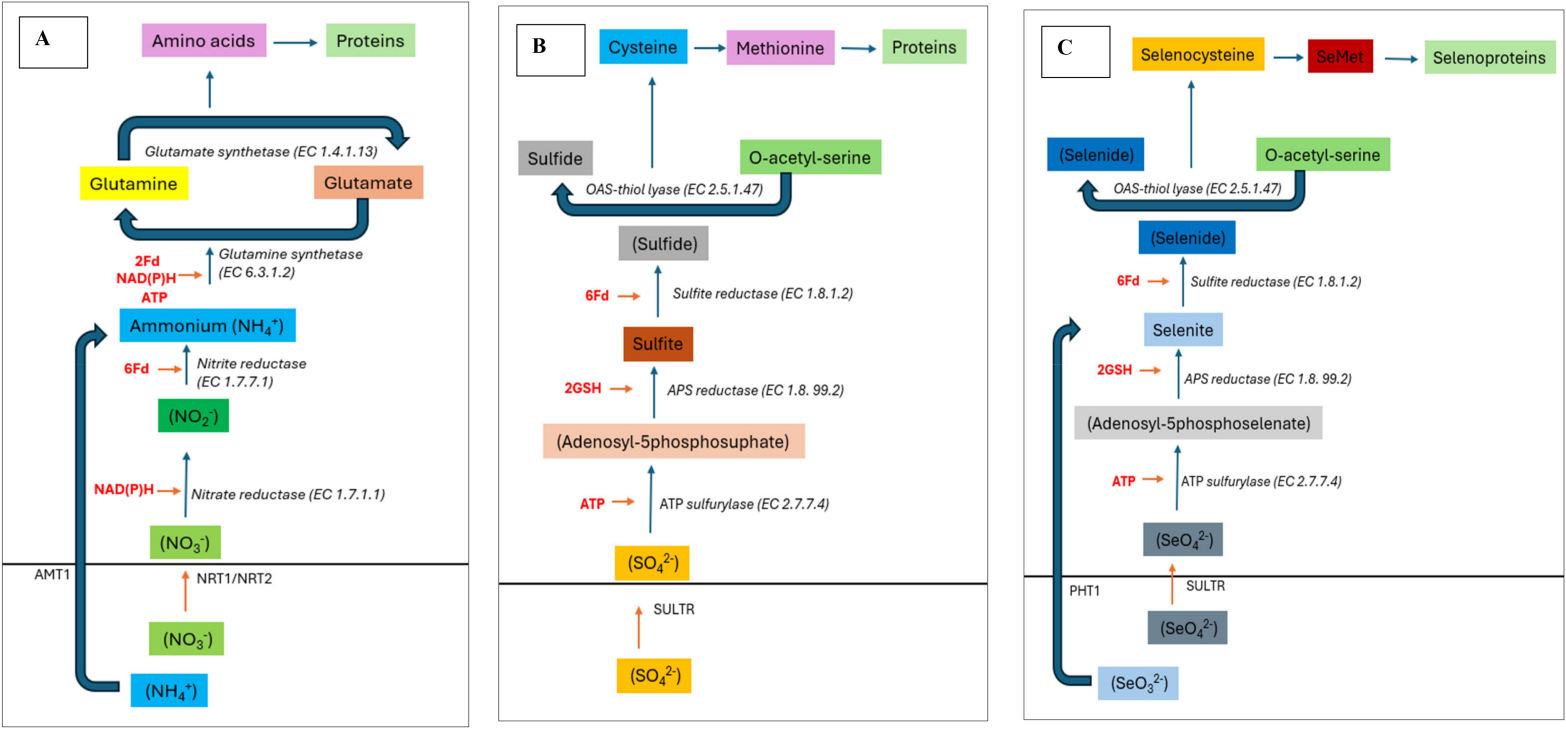
Schematic diagram showing ubiquitous nature of pathways involved in the assimilation of N, S, Se required for amino acid synthesis. **(A)** Nitrogen assimilation in roots follows sequential reduction into nitrite and ammonium which is then incorporated into amino acids. **(B)** Sulphur assimilation also follows same sequential reduction into sulfite and sulfide before synthesis of S-containing amino acids primarily cysteine and methionine. Lastly, **(C)** Selenium assimilation follows the sequential reduction into selenite, selenide and finally used to synthesize selenocysteine and selenomethionine which in-turn is incorporated into selenoproteins.

The review reports recent advancements in Se biofortification technologies in horticultural crops and currently both foliar and soil-based methods are in practice. Selenate is the preferential Se source taken up by the crops cultivated in alkaline soils and roots uptake it through the SULTR transporters. By contrast, crops grown on the acidic soils get benefit from selenite, as a Se source which enters the roots through *PHT* transporters (competes with P). Future research should focus on how we can improve the Se uptake in horticultural plants without interfering with S and/or P metabolism (**Figure 2**). Additionally, various crops exhibit differential Se uptake capacity/assimilation into the organic molecules which require further investigation. Lastly, it is also not clearly established that how plant roots respond to inorganic Se (Se-salts), organic-Se and nano-selenium which will improve our understanding and pave the way for crop specific Se-biofortification targets.
